# Synaptically Released Matrix Metalloproteinase Activity in Control of Structural Plasticity and the Cell Surface Distribution of GluA1-AMPA Receptors

**DOI:** 10.1371/journal.pone.0098274

**Published:** 2014-05-22

**Authors:** Zsuzsanna Szepesi, Eric Hosy, Blazej Ruszczycki, Monika Bijata, Marta Pyskaty, Arthur Bikbaev, Martin Heine, Daniel Choquet, Leszek Kaczmarek, Jakub Wlodarczyk

**Affiliations:** 1 Department of Molecular and Cellular Neurobiology, Nencki Institute, Warsaw, Poland; 2 Dynamic Organization and Function of Synapses, Interdisciplinary Institute for Neuroscience, Bordeaux, France; 3 Department of Neurochemistry and Molecular Biology, Leibniz Institute for Neurobiology, Magdeburg, Germany; University of Iowa, United States of America

## Abstract

Synapses are particularly prone to dynamic alterations and thus play a major role in neuronal plasticity. Dynamic excitatory synapses are located at the membranous neuronal protrusions called dendritic spines. The ability to change synaptic connections involves both alterations at the morphological level and changes in postsynaptic receptor composition. We report that endogenous matrix metalloproteinase (MMP) activity promotes the structural and functional plasticity of local synapses by its effect on glutamate receptor mobility and content. We used live imaging of cultured hippocampal neurons and quantitative morphological analysis to show that chemical long-term potentiation (cLTP) induces the permanent enlargement of a subset of small dendritic spines in an MMP-dependent manner. We also used a superresolution microscopy approach and found that spine expansion induced by cLTP was accompanied by MMP-dependent immobilization and synaptic accumulation as well as the clustering of GluA1-containing AMPA receptors. Altogether, our results reveal novel molecular and cellular mechanisms of synaptic plasticity.

## Introduction

Dendritic spines are small membranous protrusions along neuronal dendrites. The spines are the primary sites for excitatory synapses. The remarkable feature of excitatory synapses is their structural variability ranging from long, thin spines to short stubby- and mushroom-shaped spines. The cellular models of synaptic plasticity, long-term potentiation (LTP) and long-term depression (LTD), associate synaptic strength with either spine enlargement or spine shrinkage, respectively [1], [2], [3]. The structural rearrangement of preexisting spines and either the formation or loss of synapses accompany learning and memory processes [4], [5]; for review, see [1], [6]. Furthermore, alterations in dendritic spine shape, size, and density are associated with a large number of brain disorders, indicating that dendritic spines may serve as a common substrate for various neuropsychiatric conditions, including epilepsy, autism spectrum disorder, schizophrenia, addiction, and Alzheimer's disease [7], [8], [9], [10], [11], [12], [13], [14].

Dendritic spines typically consist of a head that is connected to the dendrite by a neck. The size of the spine head is proportional to the postsynaptic density (PSD) area and correlates with synaptic α-amino-3-hydroxyl-5-methyl-4-isoxazole-propionate (AMPA) receptor (AMPAR) content as well as with the synaptic strength [15], [16], [17]. Changes in synaptic efficacy are thought to underlie information coding and memory storage in the brain and might depend on the regulated trafficking of AMPARs into and out of synapses [18], [19]. Long-term potentiation relies on newly inserted AMPARs that are incorporated into synapses [20], [21], [22], [23], [24], [25], [26]. Moreover, AMPARs have been shown to underlie activity-dependent changes in excitatory synaptic function during different forms of learning, including fear conditioning [19], [27], [28], [29] and spatial learning [30], [31]. The function of synaptic receptors and ion channels can be modulated by extracellular matrix (ECM) proteases that are also able to generate molecular signals, such as products of their use-dependent proteolytic cleavage, supporting the concept of a “tetrapartite synapse” [32].

Matrix metalloproteinases (MMPs) are pericellularly acting endopeptidases that play an essential role in the dynamic remodeling of the ECM via the cleavage of numerous extracellular substrates, including growth factor precursors, cell surface receptors and adhesion molecules [33], [34], [35], [36], [37]. Several lines of evidence indicate MMPs' involvement in synaptic plasticity [38], [39], [40], [41]. In particular, MMP activity was found to be necessary for the rapid spine enlargement following theta-burst pairing (TBP) protocol that induced LTP [42]. Furthermore, locally delivered MMP-9 caused spine expansion and synaptic potentiation [42]. In hippocampal cultures or slices, exogenous MMP-9 was reported to promote the formation of either elongated, thin spines [43], [44] or solely enlarged spines [42]. However, the precise effect of endogenous MMP-9 on synaptic remodeling has remained elusive.

Herein, we significantly expand the previously reported results by documenting that endogenous MMP activity controls structural spine plasticity and AMPAR mobility in dissociated hippocampal cultures. We demonstrate that chemical LTP induces an MMP-dependent enlargement of a subset of small spines, as well as immobilization, synaptic accumulation and clustering of GluA1-containing AMPARs at dendritic spines. Our work reveals novel features of GluA1 behavior at potentiated synapses and elucidates the contribution of endogenous MMP activity to structural and functional plasticity.

## Materials and Methods

### Ethics Statement

This study was carried out in accordance with the Ethical Committee on Animal Research of the Nencki Institute, based on the Polish Act on Animal Welfare and other national laws that are in full agreement with EU directive on animal experimentation. The protocols were approved by the Committee on the Ethics of Animal Experiments of the Nencki Institute (Permit Number: 211/2011). All effort was made to minimize animal suffering.

### Preparation of dissociated neuronal cultures for live imaging

Dissociated hippocampal cultures were prepared from P0 (postnatal day 0) Wistar rats as described previously [45]. Briefly, brains were removed and hippocampi were isolated on ice in dissociation medium; DM (in mM: 81.8 Na_2_SO_4_; 30 K_2_SO_4_; 5.8 MgCl_2_; 0.25 CaCl_2_; 1 HEPES pH 7.4; 20 Glucose; 1 Kynureic Acid; 0.001% Phenol Red). Hippocampi were later incubated twice with papain solution (100 U in DM, Worthington, NY) for 15 minutes at 37°C and rinsed subsequently 3 times in DM. The digestion was stopped by incubating with trypsin inhibitor solution then hippocampi were rinsed in plating media (MEM; 10% fetal bovine serum; 1% penicillin - streptomycin). Hippocampi were triturated in plating medium until tissue chunk disappeared and the medium became cloudy. Triturated hippocampi were diluted 10 times in OptiMEM (Invitrogen) centrifuged for 10 minutes at room temperature, at 1000×g. The resulting cell pellet was suspended in plating medium. Cells were counted and plated at density 120,000 cells per 18 mm diameter coverslips (Assistant, Germany) coated with 1 mg/ml poly-D-lysine (Sigma) and 2.5 µg/ml laminin (Roche). Three hours later plating medium was replaced with 1 ml pre-warmed complete growth medium supplemented with 2% B-27; 1% Penicillin/Streptomycin; 0.5 mM Glutamine, 12.5 µM Glutamate. Cells were kept at 37°C, 5% CO_2_ in humidified incubator for 3 weeks. Cells were fed twice a week by replacing half of the culturing medium. Cells were transfected Effectene (Qiagen) or Lipofectamine 2000 Reagent (Invitrogen) according to manufacturer protocol at 7–10 days in vitro (DIV) with plasmid carrying RFP under β-actin promoter. Experiments described below were performed 18–23 DIV.

### Cell culture stimulation

A bath application of a mixture of forskolin, rolipram, and picrotoxin (all dissolved in DMSO) was applied to chemically induce LTP as described previously [46]. Cultured hippocampal neurons were incubated with 50 µM forskolin (Sigma), 0.1 µM rolipram (Sigma), and 50 µM picrotoxin (Sigma) in maintenance medium for 10 and 40 min. In some experiments, the cultures were preincubated with 25 µM GM6001 (Millipore), a general MMP inhibitor, for 30 min at 37°C, followed by cLTP induction. The total number of spines analyzed after cLTP stimulation in the presence of MMP inhibitor (GM6001) is 284. To examine whether the inhibition of MMP activity has effect on spine morphology cells were pre-incubated with GM6001, followed by an additional 10 and 40 min incubation without cLTP stimulation. The effect of inhibitor pretreatment on spine structure was studied on 388 spines.

### Live cell imaging

Live cell imaging was performed on cultured hippocampal 18–21 DIV neurons. To visualize gelatinase activity in living cultures, hippocampal neurons previously transfected with a plasmid that carried RFP under a β-actin promoter on 7–10 DIV were pre-incubated with fluorescein conjugate gelatin (DQ-gelatin; Molecular Probes) for 30 min at 37°C. Prior to imaging, the cells were mounted in a living chamber in which the temperature and CO_2_ concentration were controlled. Secondary dendrites of cultured hippocampal neurons were imaged for 10–15 min before stimulation, and cLTP was then induced by the bath application of forskolin, picrotoxin, and rolipram (n_cell_ = 5). Segments of dendrites (3 randomly chosen segments/cell) decorated with spines (∼90 spines/cell) were imaged every 5 min for 40 min of cLTP. In control experiments cells were treated with DMSO for 40 min (n_cell_ = 4). Images were acquired using the Leica TCS SP 5 confocal microscope with a PL Apo 40×/1.25 NA oil immersion objective using a 488 nm and 561 nm diode-pumped solid state lasers at 10% transmission with 1024×1024 pixel resolution. A series of z-stacks were acquired for each cell at 0.4 µm steps, with additional digital zoom that resulted in lateral resolution of 0.07 µm per pixel.

### Gelatinase assay in living neuronal culture

In order to visualize gelatinase activity in living dissociated hippocampal cultures cells were preincubated with fluorescein conjugate gelatine (40 µg/ml/well, DQ-gelatine, Molecular Probes) for 30 minutes at 37°C. This fluorescent substrate is quenched until digested by gelatinases, MMP-9 and MMP-2 in neuronal cultures. The increase in fluorescence is proportional to proteolytic activity of gelatinases which was visualised using confocal microscope.

### Live staining of GluA1-AMPARs

For selective labelling of cell surface GluA1-AMPARs cells were incubated with antibodies against the extracellular domain of GluA1 (1∶100, rabbit, Enzo Life Sciences) subunit for 15 min at 37°C in complete culture medium. The cells were later fixed with pre-warmed 4% PFA for 15 minutes. After washing the cells were blocked with PBS-BSA 1% for 30 minutes. Then samples were incubated with secondary antibody conjugated to Alexa 488 (1∶500, anti-mouse) and Alexa-647 (1∶500, anti-rabbit) for 2 hours at RT. After mounting the samples were visualized under a confocal microscopy (TCS, SP5, Leica) equipped with a 40×/1.25 NA oil immersion objective using the 488 nm Ar laser (for excitation of Alexa 488) and 633 nm HeNe laser (for excitation of Alexa 647) at a pixel resolution of 1024×1024 and 5.4 optical zoom. The Z-stacks of optical slices were acquired in 0.2 µm steps. The sum of Z-stack was analysed using ImageJ software (NIH). The intensity of the GluA1 and GluA2 immunostaining was determined using custom-written software under Phyton.

### Morhological analysis

The images of dendrites acquired in live imaging sessions were semi-automatically analyzed using the custom written software (SpineMagick software, patent no. WO/2013/021001). Only the spines protruding in the transverse direction (contained in the single image plane) that could be clearly distinguished were selected. Only the spines belonging to the secondary dendrite were chosen; the motivation for this restriction is to eliminate possible systematic differences in spine morphologies that due to the location of spines on dendrite with different ranks. The head width was defined as the diameter of the largest spine section while the bottom part of the spine (1/3 of the spine length adjacent to the dendrite) was excluded. The same spines were identified on the subsequent frames acquired in the live imaging session, which were recorded before the cLTP stimulation (with the mixture of forskolin, rolipram and picrotoxin) as well as 10 and 40 minutes after the stimulation. The total number of spines analysed after cLTP stimulation is 452 while 251 spines were examined after incubation with DMSO.

### Measure colocalization

In order to detect and calculate the level of gelatinase activity on dendritic spines, dendritic shafts as well as on the spine head of small spines the colocalization map approach was employed. In addition to the colocalization measures, which are overall quantities associated with the entire image, such as Pearson's correlation coefficient, the local information indicating the contributions of the regions of the image into overall colocalization measure is an important indicator (introduced by [47]). Such information is represented by the colocalization map. The maps were represented as the colocalization score displayed on a colour scale.

The colocalization score C (i, j) is defined in such a way that: a) it is equal zero whenever the intensity of fluorescence on either channels is zero b) it has the maximal value only if the intensities on both channels are maximal in the measurement range c) for the intermediate values of the intensities it reaches its maximum value in the case when both intensities are equal on their relative scale; the score will decrease when the intensity of fluorescence of one protein is lowered, even if the fluorescence of the second one is elevated.

### Calculation of changes in spine parameters

The systematic effects in changes of the dendritic spine morphology may be obscured by the measurement variation resulting from the diversity of spines and the spontaneous fluctuations of the spine shape [48]. In order to minimize these effects in the life-imaging analysis, the same spines were identified in the time-series of images and the relative changes (i.e. (x_1_-x_0_)/x_0_) were calculated and plotted using logarithmic scale.

### Preparation of dissociated neuronal cultures and sptPALM imaging

Hippocampal neuronal cultures were prepared from embryonic day 18 Sprague Dawley rats following a method previously described [49], [50]. On the day of seeding (0 DIV) cells were co-electroporated (4D Nucleofector) with GluA1-mEos and Homer-1-Cerulean plasmids and maintained for 18–21 DIV. Then neurons were mounted on an open chamber containing Tyrode's solution (containing in mM: 10 D-glucose, 120 NaCl, 3 KCl, 1 MgCl2, 2 CaCl2, 10 HEPES, pH = 7.4) and were observed on a Nikon inverted microscope equipped with EMCCD camera using total internal reflection geometry (TIRF). GluA1-mEos subunits of AMPARs were imaged every 5 minutes till 30 minutes after cLTP induction following a brief control (before cLTP induction) acquisition. In some experiments cells were preincubated with GM6001 for 30 minutes then cLTP was induced. GluA1 subunit of AMPARs tagged with mEos were imaged with 20 millisecond exposure time at the rate of 20 frames per second and in each recording session 3000 frames were collected. Images were acquired using high numerical aperture (100×/1.49, oil) objective to avoid inducing detrimental effect to living cells. Differential interference contrast microscopy (DIC) images were taken prior to and after excitation to serve as a reference point for cell viability.

### GluA1-mEos tracing and surface diffusion calculation

In order to resolve the dynamics of GluA1-AMPARs during cLTP in living hippocampal neurons PALM and single-particle tracking technique were combined. Information on the position of GluA1 subunits was obtained by activating, localizing and bleaching many subsets of GluA1 subunits tagged with monomeric Eos. Monomeric Eos is a photoshiftable fluorescent protein, whose fluorescence excitation and emission spectra shift following illumination. Particularly, exposure to ultraviolet or blue light (405 nm) causes an irreversible shift in its spectrum from green state to orange state. In each image of the recorded sequence, the single fluorescent GluA1 subunits appear as bright spots. For image analysis, custom-made program written in Metamorph (Molecular Devices) was used following a method previously described [51]. Briefly, the positions and position errors for each fluorescent GluA1 signal (spot) were localized by fitting two-dimensional Gaussian to the intensity patterns of the spot. Summed trajectories were drawn by linking molecular peaks of GluA1 signal in consecutive frames according their proximity (on the condition that the molecule cannot move more than 3 pixels between two adjacent frames). Diffusion analysis was performed on the trajectories with at least 8 points (molecule was detected in 8 consecutive frames).

Mean-square displacement (MSD), for time interval (t = nΔT) and for the trajectory of N data points, was calculated for each trajectory using the standard formula:
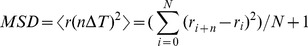



MSD is average squared distance travelled by molecule in unit of time. For each trajectory diffusion coefficient was calculated from linear fit of MSD = 4Dt. Diffusion coefficient characterizes how far GluA1-AMPARs move from the position at t = 0 time point in a given time interval.

### Methods for MEA experiments

#### Preparation of dissociated neuronal cultures

All experimental procedures were carried out in accordance with the EU Council Directive 86/609/EEC and were approved and authorised by the local Committee for Ethics and Animal Research (Landesverwaltungsamt Halle, Germany). For multichannel recordings, hippocampi of Wistar rat embryos at gestation day 18 (E18) were isolated as described previously [52], and cell suspension (∼750,000 cells/mL) was plated onto 60-channel microelectrode arrays (MultiChannel Systems, Reutlingen, Germany) pre-coated with Poly-D-lysine. After plating, all cultures were maintained in serum-free Neurobasal medium at 37°C in a humidified atmosphere (95% air and 5% CO_2_). Culture medium has been partially exchanged with conditioned medium on a regular basis once a week.

#### Multichannel recordings of the network activity and analysis of data

The extracellular neuronal activity prior to and after induction of cLTP was recorded (sampling rate 10 kHz, MEA1060BC-INV; MultiChannel Systems, Reutlingen, Germany) in cultures at DIV21–25 under conditions (temperature, humidity, gas composition) identical to those during incubation period. The offline analysis was carried out on 600-sec long sessions at each time-point for each culture and included threshold-based (±6 SD computed on spike-free interval) spike detection in high-pass (300 Hz) filtered records, followed by identification of bursts (≥ 5 spikes with inter-spike interval <100 ms). In order to evaluate cLTP-induced changes in the network activity, the mean firing and bursting rates, as well as mean values for burst duration and the mean number of spikes per burst were calculated separately for each active channel in each culture and compared to respective mean values obtained during baseline. All analyses of multi-channel recordings were carried out using Spike2 software (Cambridge Electronic Design, Cambridge, UK).

### Statistical analysis

The statistical values are expressed as mean ± standard error of the mean (SEM). Datasets were tested using the two-tailed Student's t-test and if the number of groups were larger than two one-way ANOVA was used. Groups were compared using Mann-Whitney test in analysis of GluA1 clustering. The statistical analyses were performed using Origin 8. Indications of significance correspond to *p*<0.05 (*), *p*<0.01 (**), and *p*<0.001 (***).

## Results

### cLTP affects the morphology of small spines in an MMP-dependent manner

Chemical LTP evoked by cAMP analogue, as a model system, was initially described in acute hippocampal slices [46]. Then it was successfully applied for dissociated hippocampal neuronal cultures [53], [54]. Recently, Niedringhaus et al. (2012) have demonstrated an enhancement of MMP-dependent neuronal activity within *in vitro* networks of hippocampal neurons subjected to cLTP protocol.

**Figure 1:**
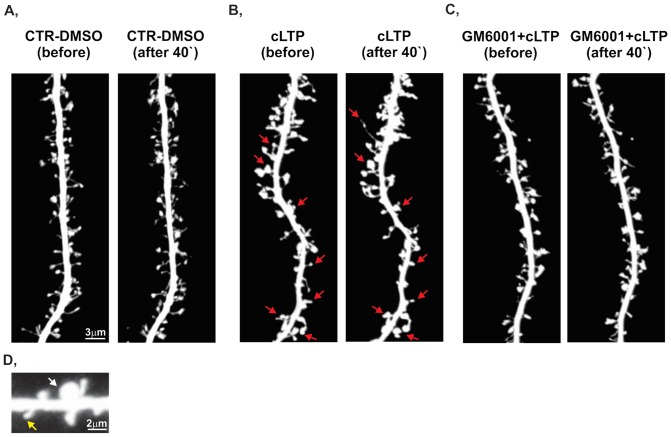
cLTP induces MMP-dependent changes in dendritic spine morphology. Representative images of RFP-transfected dendrites from dissociated hippocampal cultures (21 DIV) were captured during a live imaging session under control, cLTP-stimulated, and GM6001+cLTP-stimulated conditions. (**A**) Incubation with DMSO (as a compound-free solvent) did not induce prominent alterations in spine morphology. (**B**) Forty minutes of cLTP stimulation caused spine enlargement compared with controls (i.e., cultures before cLTP induction). The arrows indicate the locations of spines that underwent structural plasticity, including spine enlargement and the formation of spine head protrusion. (**C**) The inhibition of MMP activity by GM6001 (25 µM) abolished cLTP-induced morphological changes in spine morphology.(D) Typical examples of small and large dendritic spines are shown.

In our initial experiments, we confirmed that treatment with rolipram, forskolin, and picrotoxin (i.e., the cLTP protocol used in our work, see Otmakhov et al., 2004) produced lasting (up to several hours) enhancement of network activity, reflected by increases in both spiking and bursting activity (Fig. S1). We then investigated whether such treatment affects the morphology of dendritic spines. Furthermore, because Niedringhaus et al. (2012) [55] demonstrated that the changes in network activity require metalloproteinases, we also applied a broad-spectrum MMP inhibitor, GM6001 (25 µM), to investigate the effect of MMPs on spine morphology. Figure 1 shows a short stretch of live dendrites in the control (Fig. 1A), cLTP-induced (Fig. 1B), and GM6001-pretreated (Fig. 1C) groups. Forty-minute exposure to cLTP protocol induced a prominent increase in spine area and the formation of spine head protrusions (marked with arrows in Fig. 1B, see [56]), whereas the inhibition of MMP activity prevented the cLTP-induced changes in spine morphology (Fig. 1C). Notably, cLTP did not affect spine density during the 40 min live imaging observation session (Fig. S2). Under control conditions, the spine density was 0.82±0.08 (number of protrusions per µm^2^ [mean ± SEM]). With 40 min of cLTP, the spine density was found to be 0.87±0.09. When the cLTP protocol was applied in the presence of GM6001, the spine density was 0.83±0.07.

To further characterize the influence of cLTP on spine size, relative changes in the spine head width of individual spines were quantified. We identified the same spines before and 10 and 40 min after cLTP stimulation in images captured during the live imaging session. Because the initial size of individual spines might determine the extent of synaptic strength and structural plasticity during LTP [57], [58] we divided the spines into two subpopulations (i.e., large and small spines) according to the mean of the spine area distribution. Dendritic spine was considered as small spine if its area was found to be <0,5 µm^2^. The percentage of small spines in hippocampal cultures at 21days *in vitro* was found to be app. 37%. Dentritic spines with area >0.5 µm^2^ was considered as a large spine. Representative small and large spines are shown on Fig.1D.

**Figure 2:**
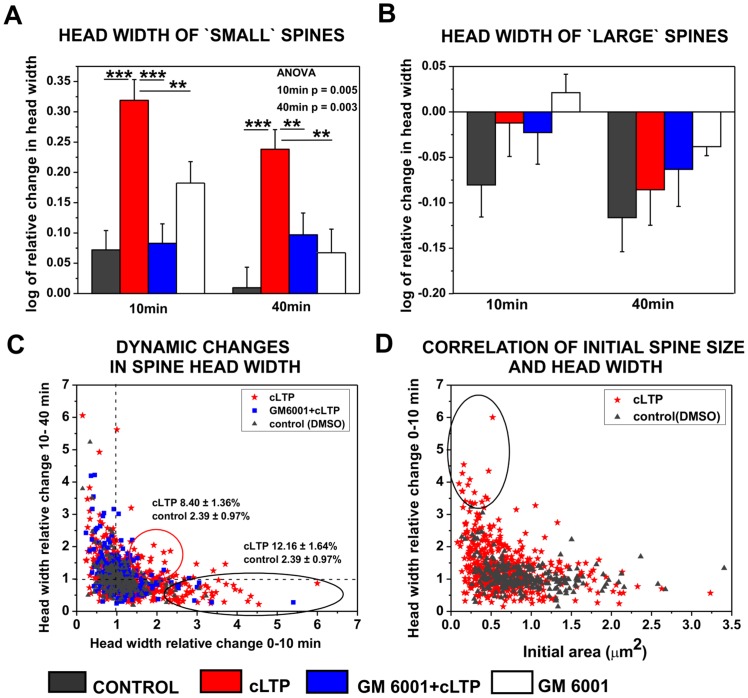
cLTP induced MMP-dependent spine growth of small spines. (**A**) Relative changes (mean ± SEM) in the spine head width of small spines after 10 and 40 min of cLTP in the presence or absence of GM6001 and after incubation with GM6001 in un-stimulated condition. (**B**) Relative changes (mean ± SEM) in the spine head width of large spines 10 and 40 min following cLTP induction in the presence or absence of GM6001 and after incubation with GM6001 in un-stimulated condition. (**C**) Correlation between relative changes in head width during 0–10 min (x-axis) and 10-40 min (y-axis) of cLTP. Groups of spines that exhibited a fast response and an increased head width during only the first 10 min are marked with a black circle. A subpopulation of spines exhibited a persistent enhancement of head width during 40 min of cLTP (marked with red circle). The numbers represent the mean ± SEM percentage of spines within the marked subpopulation. (**D**) Correlation plot of relative changes in head width (mean ± SEM) in course of spine area. Small spines displayed rapid and marked relative changes in head width (marked by circle) in response to cLTP compared with large spines.

The detailed quantification of the spine morphology of individual spines affected by cLTP revealed a robust and persistent increase in the relative spine head width of initially small spines in response to 10 min (n_spine_ = 271; 0.32±0.03, *p*<0.001) and 40 min (0.24±0.03, *p*<0.001) cLTP compared with controls (10 min: n_spine_ = 150; 0.07±0.03; 40 min: 0.01±0.03; Fig. 2A). The GM6001-induced inhibition of MMP activity abolished the enlargement of spine heads induced by cLTP. (10 min, n_spine_ = 176; 0.08±0.03, *p*<0.001 40 min 0.09±0.04, *p* = 0.005). Incubation with GM6001 had no effect on spine head width under un-stimulated condition. One-way ANOVA revealed statistically significant differences between groups of mean head width at 10 and 40 min of cLTP stimulation. The corresponding values were found to be p<0.01.

The large spines exhibited no statistically significant changes in spine head width in response to either 10 min (n_spine_ = 181; −0.01±0.04) or 40 min (–0.09±0.04) cLTP compared with controls (10 min: n_spine_ = 101; −0.08±0.04, *p* = 0.22, 40 min: −0.12±0.04, *p* = 0.61) and the GM6001-pretreated groups (n_spine_ = 111; 10 min: −0.02±0.03, *p* = 0.85, 40 min: −0.06±0.04, *p* = 0.71, Fig. 2B). Similarly, the inhibition of MMP activity by GM6001 had no effect on spine head width under control condition. Our observations indicate that cLTP stimulation led to the transformation of small spines into large, mushroom-shaped spines, and this spine growth was mediated by MMP activity.

To characterize the dynamics of spine size during cLTP, we correlated relative changes in spine head width during 0–10 min and 10–40 min of cLTP. This approach led to the identification of spine groups that displayed different dynamics of morphological changes (Fig. 2C). We found a subpopulation of spines that exhibited an increase in spine head width exclusively during 0–10 min of cLTP (12.16±1.64% of the total population). We also identified spines that exhibited a continuous enlargement of head width during 40 min of cLTP (8.40±1.36% of the total population). The blockade of MMP activity by GM6001 (25 µM) prevented the cLTP-induced increases in spine head width observed in both of these subpopulations of spines. By correlating the initial spine area with the relative changes in head width, we found that initially small spines (area ≤0.5 µm^2^) displayed a pronounced increase within the first 10 min of cLTP (Fig. 2D). Our observations indicate that cLTP induced the MMP-dependent growth of a subpopulation of small spines.

### cLTP enhances gelatinase activity at dendritic spines

**Figure 3:**
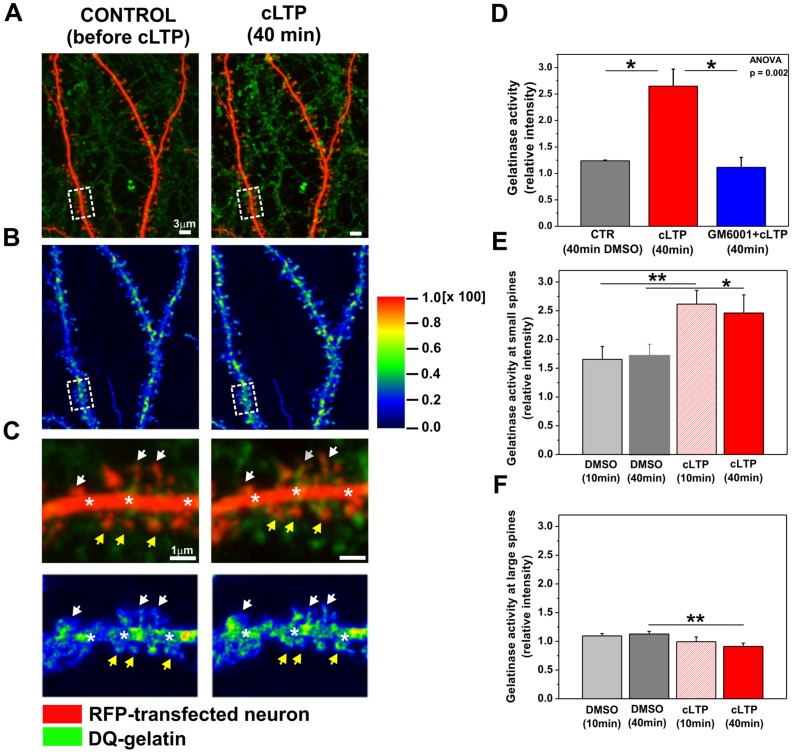
cLTP increased gelatinase (MMP-9 and MMP-2) activity at dendritic spines. (**A**) Red fluorescent protein-transfected hippocampal neurons (red channel; 21 DIV) incubated in media with 40 µg/ml/well DQ-gelatin (green channel) for 30 min prior to cLTP induction were imaged. Examples of living dendrites are shown before and 40 min after cLTP stimulation. Chemical LTP stimulation elevated gelatinase activity compared with controls (i.e., before cLTP). The enhancement of gelatinase activity was localized in close proximity to dendritic spines. (**B**) Colocalization maps. The color map shows the increase in colocalization between gelatinase activity and dendritic segments before and 40 min after cLTP induction. The colors on the image correspond to the color-coded scale bar. Regions with the highest contribution to colocalization were localized on dendritic shafts and spines. (**C**) A short stretch of dendrite and the corresponding colocalization map are shown at higher magnification (marked with rectangles in Fig. 3A and B). Elevated gelatinase activity was detected on dendritic shafts (marked with stars) and small spines (marked with yellow arrows) at 40 min of cLTP compared with controls (i.e., before cLTP). (**D**) Quantification of gelatinase activity along neuronal dendrites under control, cLTP-stimulated, and GM6001+cLTP-stimulated conditions. Relative intensities ([f–f_0_]_cLTP_/[f–f_0_]_control_) are presented. (**E**) Relative changes ([f–f_0_]_cLTP_/[f–f_0_]_control_) in gelatinase activity localized at spine heads of small, growing spines are shown under control and cLTP-stimulated conditions. (**F**) Relative changes ([f–f_0_]_cLTP_/[f–f_0_]_control_) in gelatinase activity localized at large, not-growing spines are plotted under control and cLTP-stimulated conditions. Bar plots of the mean intensities (mean ± SEM) are shown.

Our previous experiments indicated a role for MMP activity in the mediation of cLTP-driven changes in spine size. We also reported that the cLTP protocol reported herein selectively upregulated MMP-9 gelatinase activity at 10 and 40 min [56]. To subcellularly localize endogenous gelatinase activity, RFP-transfected cultures were exposed to DQ-gelatin, a fluorogenic substrate for gelatinases, and cLTP was then induced. Time-lapse images of live individual spines on secondary and tertiary dendrites were acquired at 5 min intervals over 40 min of cLTP stimulation. We observed an enhancement of gelatinase activity throughout the culture in response to cLTP, indicating its widespread enhanced production by cultured cells (Fig. 3A). Analysis of its cellular expression revealed co-localization between the fluorescence signal of cleaved gelatin and RFP-expressing neuronal dendrites. Figure 3A shows a short stretch of dendrites of RFP-expressing hippocampal neurons (red channel) incubated with DQ-gelatin (green channel) before and 40 min after cLTP stimulation. The detailed co-localization study revealed that the enhanced gelatinase activity induced by cLTP stimulation was mainly localized at dendritic spines and shafts. Figure 3B shows a quantitative co-localization map before and 40 min after cLTP stimulation. Higher-magnification images of Fig. 3A and B are shown in Fig. 3C. Figure 3C demonstrates differences in gelatinase activity between small and large spines after cLTP stimulation. Enhanced gelatinase activity induced by cLTP was localized at small spines (yellow arrows) and dendritic shafts (stars).

Green fluorescence intensity, which is proportional to gelatinase activity, was then quantified at the sites of co-localization with 40 min of cLTP stimulation. To confirm that the enhancement of gelatinase activity at the spines upon cLTP was specific to MMP activity, hippocampal neurons were incubated with GM6001 (25 µM) 30 min prior to cLTP stimulation. Relative changes in gelatinase activity were calculated in control, cLTP-stimulated, and GM6001-pretreated conditions (Fig. 3D). Statistically significant increase in gelatinase activity (green fluorescence intensity relative to control) was observed at 40 min of cLTP (2.65±0.32, *p* = 0.05) compared with controls (1.24±0.01). The stimulation of hippocampal neurons in the presence of GM6001 prevented the cLTP-induced increase in gelatinase activity (1.11±0.19, p = 0.02). One-way ANOVA revealed a statistically significant increase between groups of gelatinase activity (p<0.01). These observations indicate that cLTP induced by forskolin, rolipram, and picrotoxin elevated gelatinase activity at the dendritic spines and shafts of live hippocampal neurons.

To determine whether growing spines specifically exhibit enhanced gelatinase activity upon cLTP induction, we quantified the relative changes in green fluorescence intensity (which is proportional to gelatinase activity) at spines with increased head width with 10 and 40 min of cLTP (Fig. 3E). Strikingly, we found that 58% of the small spines exhibited an increase in head width in association with enhanced gelatinase activity with 10 min of cLTP induction. The increase in gelatinase activity at 10 min (2.61±0.24, *p*<0.005) was found to be statistically significant compared with controls (i.e., DMSO-treated cells; 1.65±0.22). With 40 min of cLTP stimulation, 71% of growing spines co-localized with increased gelatinase activity. Student's *t*-test revealed a statistically significant increase in gelatinase activity with 40 min of cLTP (2.46±0.32, *p*<0.05) at growing spines compared with controls (1.71±0.20). We also quantified the relative changes in gelatinase activity at large spines after cLTP stimulation (Fig. 3F). We observed no alterations at 10 min (DMSO; 1.09±0.04, cLTP; 0.99±0.08, *p* = 0.27) while a significant decrease in gelatinase activity was detected after 40 min cLTP (DMSO; 1.13±0.05; cLTP; 0.91±0.06, *p* = 0.006). Our observations clarify that enhanced gelatinase activity solely promoted the growth of small spines of hippocampal neurons upon cLTP.

### MMPs contribute to synaptic recruitment and immobilization of GluA1-AMPARs

**Figure 4:**
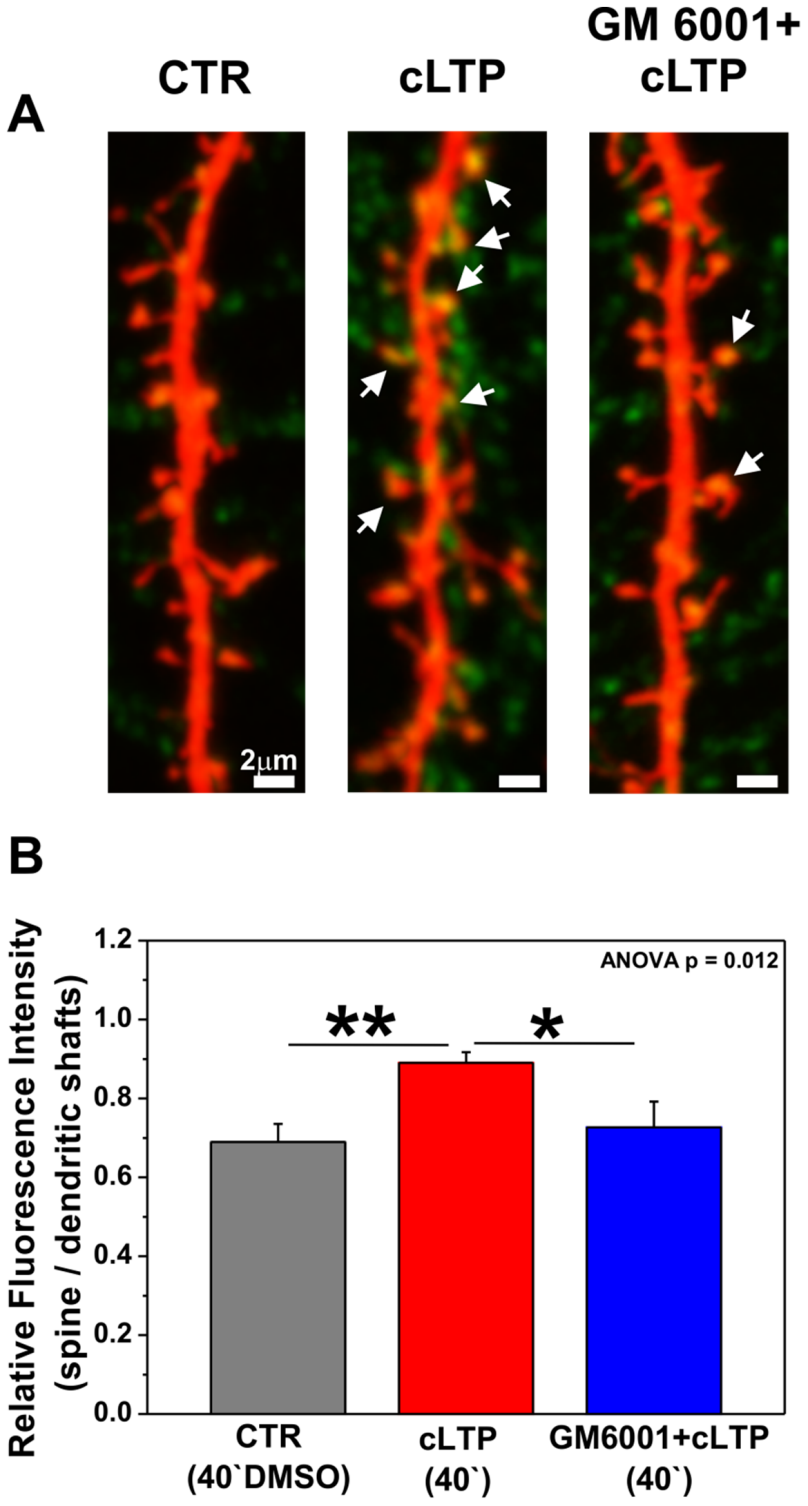
cLTP drives GluA1-AMPARs into dendritic spines in an MMP-dependent manner. (**A**) RFP-transfected hippocampal neurons (21 DIV) were stimulated with DMSO (CTR) or the cLTP mixture in the presence or absence of GM6001 (25 µM) and immunolabeled for the GluA1 subunit of AMPARs. The arrows show the enhancement of GluA1 immunostaining (green channel) at dendritic spines 40 min after cLTP stimulation. (**B**) Ratio of fluorescence intensities of GluA1 immunolabeling (mean ± SEM) at spines *vs*. dendritic shafts under control, cLTP-stimulated, and GM6001+cLTP-stimulated conditions.

Chemical LTP, similar to other forms of LTP, was found to involve the enhanced synaptic expression and immobilization of AMPARs [23], [59]. To examine whether MMP-dependent spine enlargement induced by cLTP associates with increased number of synaptic AMPARs, we immunolabeled the cell surface GluA1-AMPAR subunits in live hippocampal neurons (Fig. 4A). In order to visualize neuronal dendrites and dendritic spines cell were transfected with RFP prior to GluA1 immunostaining. Forty minutes of cLTP treatment increased GluA1-AMPARs content compared with controls, whereas pretreatment with GM6001 (25 µM) prior to the cLTP protocol decreased GluA1 immunoreactivity. The cLTP-induced enhancement of GluA1 immunoreactivity (green channel) was found to be localized at dendritic spines (marked with arrows in Fig. 4). Note, that GluA1 immunostaining derived from not RFP-transfected, surrounding neurons is also present in mature hippocampal culture (21DIV).

Next, the relative distributions (i.e., fluorescent readouts that are proportional to the amount of endogenous GluA1 subunits) were calculated within dendritic spines *vs*. dendritic shafts (Fig. 4B). We observed a statistically significant increase in endogenous GluA1 subunit content (0.89±0.03, *p* = 0.002, *n*_cell_ = 13) at dendritic spines with 40 min of cLTP induction compared with controls (i.e., DMSO-treated cells; 0.68±0.05, *n*_cell_ = 9), reflected by relative changes in optical density (OD_spine_/OD_shaft_). To reveal the contribution of MMP activity to the cell-surface accumulation of AMPARs, hippocampal neurons were preincubated with GM6001 (25 µM) prior to cLTP induction. The blockade of MMP activity by GM6001 resulted in a statistically significant decrease in the abundance GluA1 subunits at dendritic spines (0.72±0.07, *p* = 0.049, *n*_cell_ = 7). One-way ANOVA revealed statistically significant differences between groups of relative fluorescent intensity (OD_spine_/OD_shaft_, p<0.05). These results demonstrate that the proteolytic activity of MMPs is required for the recruitment of GluA1-containing AMPARs at spines during cLTP.

To explore the role of gelatinases (MMP-9 and MMP-2) in AMPAR surface diffusion during cLTP, we first overexpressed GluA1 subunits labeled with monomeric Eos fluorescent protein together with Homer-cerulean, a postsynaptic marker, and traced AMPAR surface diffusion using a single-particle-trafficking photoactivated localization microscope (sptPALM). The trajectories of single GluA1 molecules were then constructed from a series of images acquired at the rate of 20 Hz, and the instantaneous diffusion coefficient (D; µm^2^/s) was calculated.

**Figure 5:**
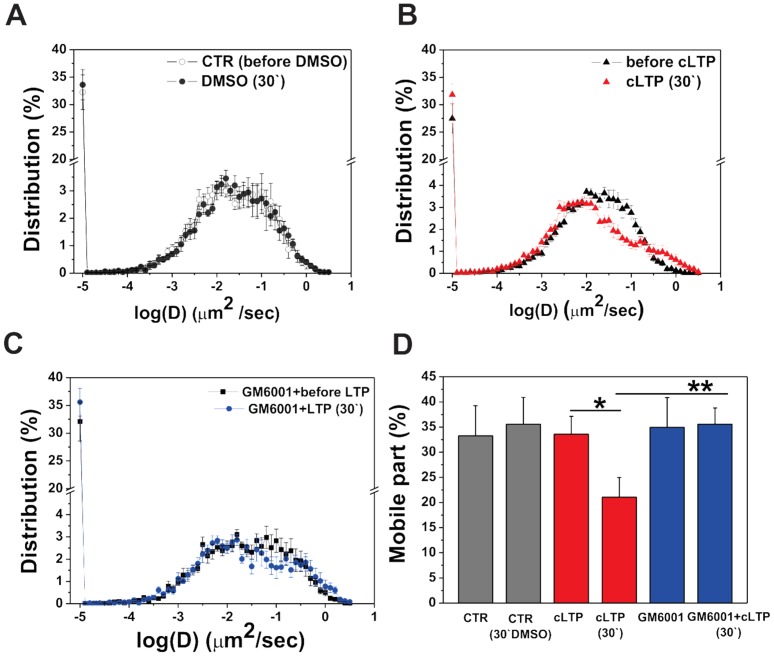
MMPs' activity influences the surface diffusion of GluA1-AMPARs during cLTP. Dissociated hippocampal neurons (7 DIV) were co-transfected with GluA1-mEos and the postsynaptic marker of Homer-1-cerulean, and cLTP was then induced at 21 DIV with or without GM6001. The surface diffusion of GluA1 was tracked using sptPALM. The global diffusion coefficient was calculated for 25,000 trajectories per condition. (**A**) Distribution of global diffusion coefficients of the GluA1 subunit after 30 min incubation with DMSO (i.e., the solvent for forskolin, rolipram, and picrotoxin). (**B**) Distribution of global diffusion coefficients of GluA1 before and after 30 min of cLTP stimulation. (**C**) Distribution of global diffusion coefficients obtained from GluA1-mEos before and after 30 min of cLTP in the presence of GM6001 (25 µM). The inhibition of MMP activity eliminated cLTP-induced GluA1 immobilization. (**D**) Mean ± SEM percentage of mobile fraction of GluA1-AMPARs under the indicated conditions.

Cultured hippocampal neurons were incubated with either RFP or DMSO (as a control), and GluA1-AMPAR diffusion was recorded at 5 min intervals over a total of 30 min of cLTP stimulation. We found that incubation with DMSO had no effect on global GluA1-AMPAR diffusion (Fig. 5A). In contrast, global GluA1-AMPAR diffusion was strongly decreased with 30 min of cLTP compared with the control condition (i.e., before cLTP stimulation). The distribution (D; µm^2^/s) of GluA1 subunits was found to be restricted to lower values with 30 min of cLTP, whereas the distribution (D; µm^2^/s) had a subpopulation at higher values before cLTP stimulation (Fig. 5B). To determine the involvement of MMPs in GluA1-AMPAR lateral diffusion, we used GM6001 pretreatment prior to cLTP stimulation, and GluA1-AMPAR diffusion was traced. We found that the blockade of MMPs activity by GM6001 (25 µM) abrogated the cLTP-induced decrease in global GluA1-AMPAR mobility (Fig. 5C).

Furthermore, we plotted the changes in the percentage of mobile GluA1-AMPARs in control, cLTP-treated, and GM6001-pretreated cells and made comparisons between conditions (Fig. 5D). We found that cLTP stimulation significantly decreased the percentage of globally mobile GluA1-AMPARs at 30 min of cLTP (21.06±3.93%, *p* = 0.03) compared with the control condition (i.e., before cLTP stimulation; 33.59±3.56%). Pretreatment with GM6001 abolished the cLTP-induced immobilization of GluA1-AMPARs (35.58±3.20%, *p* = 0.01) compared with cLTP-stimulated cells and brought the globally mobile GluA1-AMPAR to control levels (before DMSO: 33.26±5.99%; after 30 min incubation with DMSO: 35.58±5.32%). Incubation with GM6001 without cLTP induction did not affect the global mobility of GluA1-AMPARs (data not shown).

**Figure 6:**
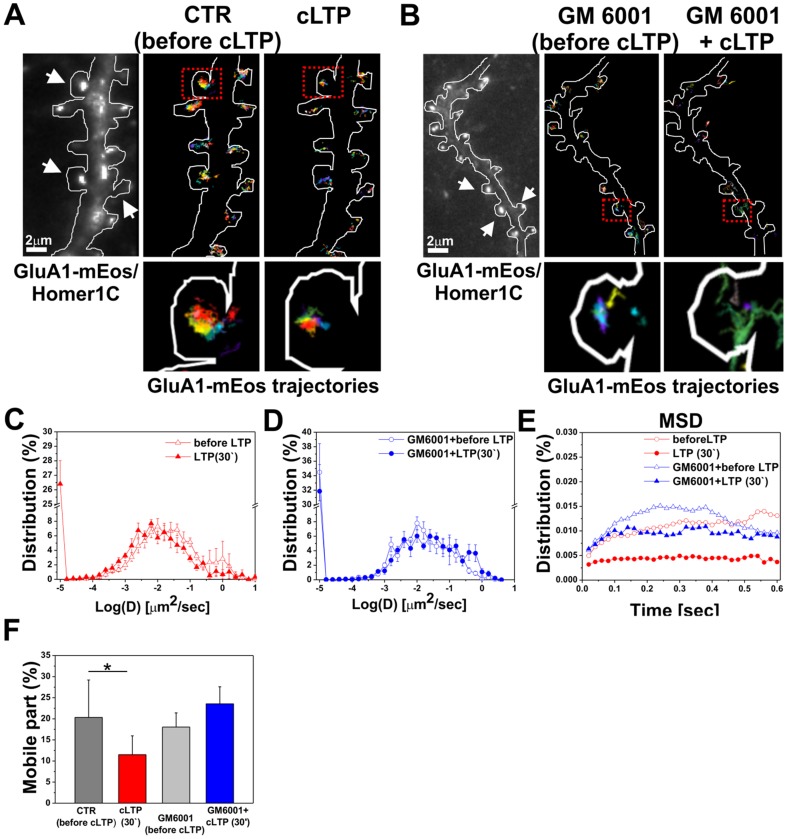
MMP localization and activity affect surface mobility of GluA1 at synapses during cLTP. (**A, B**) Imaging of GluA1-mEos in live cultured neurons. The trajectories of GluA1-containing AMPARs on dendrites of 21 DIV cultured GluA1-mEos/Homer1C-co-transfected hippocampal neurons are shown under the indicated conditions. Imaged dendritic segments are shown. Postsynaptic sites accumulated GluA1 and Homer1C (arrows). High-magnification images of dendritic spines show more confined synaptic trajectories of GluA1 after cLTP stimulation compared with the control and GM6001-pretreated (**C, D**) Distribution of the instantaneous diffusion coefficients of synaptic trajectories obtained from GluA1-mEos before and after 30 min of cLTP stimulation in the absence or presence of GM6001 (25 µM). (**E**) Distribution of mean square displacement of GluA1-mEos at synapses as a function of time under the indicated conditions. (**F**) Mean ± SEM percentage of synaptically mobile GluA1-containing AMPARs before and 30 min after cLTP induction in the presence or absence of GM6001.

Figure 6A and B show short segments of GluA1-mEos/Homer 1-co-transfected dendrites with single trajectories of GluA1 subunits at synapses before and after 30 min of cLTP stimulation with or without GM6001. High-magnification images of dendritic spines show more confined synaptic trajectories of GluA1 after cLTP stimulation compared with the control and GM6001-pretreated groups. The diffusion coefficient of GluA1-AMPARs was strongly reduced at synapses marked with Homer, whereas the inhibition of MMP activity by GM6001 abolished the decrease in the surface diffusion of GluA1-AMPARs at synapses (Fig. 6C, D). In parallel, synaptic mean square displacement (MSD) analysis showed a three-fold decrease in MSD after cLTP compared with controls (i.e., before cLTP; Fig. 6E). Consistent with this finding, the percentage of the mobile fraction of GluA1-AMPARs at synapses significantly decreased after cLTP stimulation (11.48±4.48%, *p* = 0.048, Student's *t*-test) compared with controls (i.e., before cLTP stimulation; 20.34±8.87%). The inhibition of MMP activity by GM6001 eliminated cLTP-induced GluA1-AMPAR immobilization at synapses (GM6001 + before cLTP; 18.05±3.36%; GM6001 +30 min cLTP: 23.55±4.04%). The more confined GluA1 mobility after cLTP indicates that GluA1-AMPARs were anchored/trapped in synaptic membranes after the induction of cLTP, and active MMPs promoted AMPAR immobilization at synapses in response to the plasticity stimulus.

### MMPs facilitate GluA1-AMPAR accumulation at synapses

**Figure 7:**
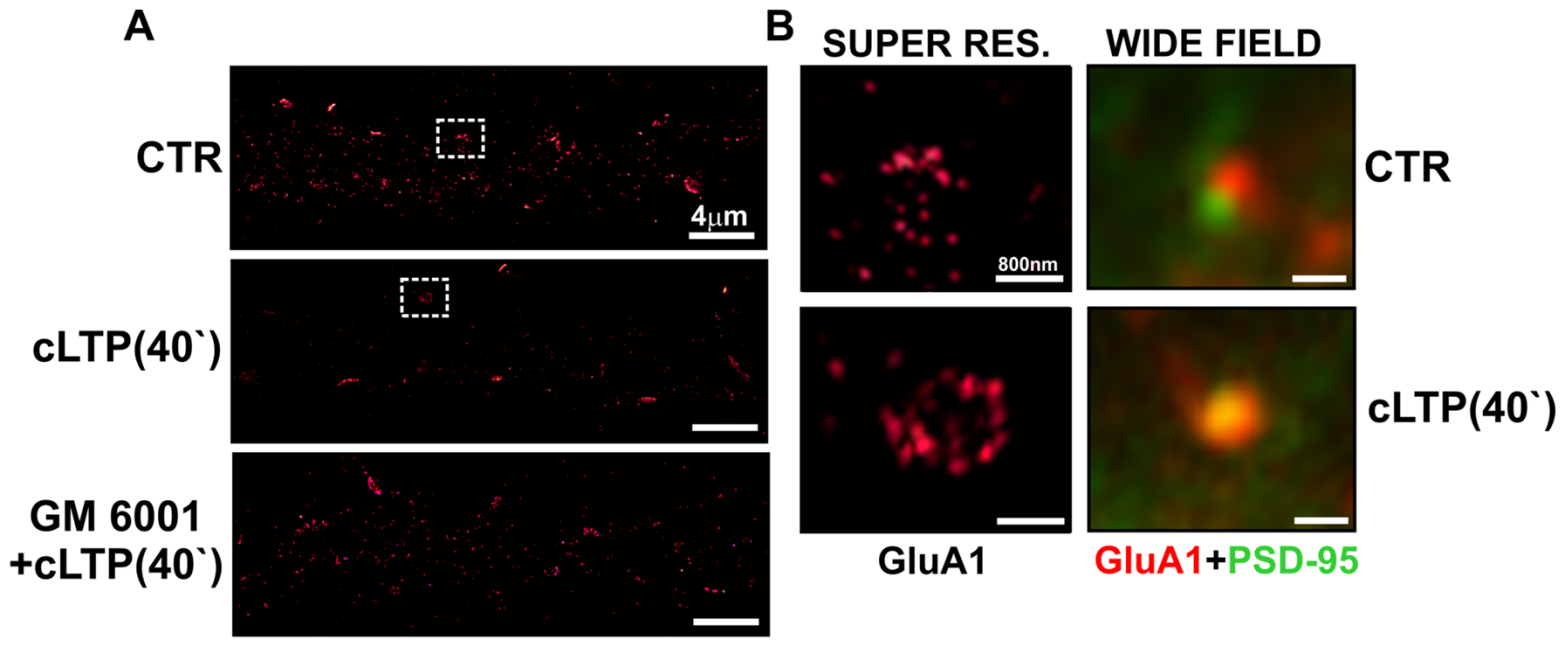
Chemical LTP induces clustering of synaptic GluA1-AMPARs in an MMP-dependent manner. Hippocampal neurons (21 DIV) were treated with DMSO (CTR) or forskolin, rolipram, and picrotoxin (cLTP stimulation) with or without GM6001 (25 µm) and then immunolabeled for the GluA1 subunit of AMPARs and the postsynaptic marker of PSD-95. Clusters of GluA1-containing AMPARs were imaged using a ground-state depletion microscope (GSDM). (**A**) GluA1-AMPAR staining under control conditions and after cLTP stimulation with or without GM6001. (**B**) Comparison of GluA1-AMPAR clusters at PSD-95-positive synapses using GSDM and a wide-field fluorescent microscope.

Finally, we examined the distribution of synaptic GluA1-AMPARs in cLTP-treated hippocampal cultures (21 days *in vitro* [DIV]) using a supperresolution microscopy approach. Endogenous GluA1 levels were assayed by immunofluorescence using a GluA1 subunit-specific antibody directed against the extracellular domain of GluA1. PSD-95 immunolabeling was used to identify glutamatergic synapses. To visualize GluA1-containing AMPARs, neuronal dendrites were imaged using a ground state depletion microscope (GSDM; Fig. 7). We found that cLTP stimulation led to an increase of GluA1- AMPAR content at PSD-95-positive synapses. Following cLTP stimulation, the number of GluA1-containing AMPAR clusters per 16 µm^2^ dendrites increased to 11.91±2.70 (*p* = 0.04) over control levels (6.40±0.40). Strikingly, the inhibition of MMP activity by GM6001 pretreatment (25 µM) prior to cLTP induction prevented the accumulation of GluA1-AMPARs at synapses. Following GM6001 pretreatment the number of GluA1 clusters (7.84±0.96) did not significantly differ from controls.

## Discussion

In the present study, we show that a chemically induced form of LTP (cLTP) involves the enlargement of a subset of small dendritic spines (ca. 10% of the total spine number), concomitantly with the immobilization and synaptic accumulation of GluA1-containing AMPARs. All of those phenomena require the enzymatic activity of MMPs. Furthermore, cLTP increases MMP-9 (gelatinase B) activity, which is also observed at dendritic spines that are undergoing remodeling. These data suggest a novel, MMP- dependent, molecular mechanism of the synaptic plasticity.

We drove bursting activity in a network of cultured hippocampal neurons to produce robust and steady synaptic enhancement that mimicked LTP. Chemically induced LTP in cultured neurons shares many key properties with the electrically induced LTP of CA1 neurons in hippocampal slices (for review, see [53]). The cLTP model used in the present study was reported to be associated with enhanced neuronal network activity of cultured hippocampal neurons [55], with the extrasynaptic delivery of GluA1-containing AMPARs [54] and enhanced MMP-9 activity [56]. Furthermore, Szepesi et al. (2013) reported that cLTP evoked formation of spine head protrusions in MMP-9 dependent manner. However, the previous studies with this model neither addressed the spine head size nor the AMPARs' mobility at the synapses. Those issues are of pivotal importance for the synaptic plasticity.

### cLTP induces spine growth in a MMP-dependent manner

To assay spine morphology, we monitored the head widths of individual spines targeted by cLTP in live hippocampal neurons and concomitantly measured gelatinase (MMP-9 and MMP-2) activity. We have shown that changes in gelatinase activity are localized predominantly on small spines. The findings that gelatinase activity is localized on small spines can be explained by previous findings that MMP-9 protein predominantly present on the small spines [60] and thus can be released preferentially from them to reveal local gelatinolytic activity. We showed that cLTP induced robust and permanent spine enlargement. We demonstrated that small (i.e., immature) spines exhibited a significant increase in spine head width, whereas large (i.e., mature) spines displayed no remarkable changes in head width upon cLTP. The growth of small spines in response to cLTP suggests that these are the preferential sites for cLTP induction.

The selective enlargement of small spines upon either cLTP or glutamate uncaging was previously demonstrated in hippocampal slices and slice cultures [61], . Additionally, larger postsynaptic densities (i.e., larger head widths) were also found in certain spine populations after tetanic stimulation in hippocampal slices [63]. In the present study, we found that dendritic spines displayed either fast and robust head width enlargement within 0–10 min of stimulation or a less prominent but persistent increase with 10–40 min of cLTP. Consistent with other studies, only a small percentage of spines (∼10%) were found to be enlarged upon cLTP. Considering that the spine enlargement concerned mainly those that expressed enhanced gelatinase activity, we propose that our data provide a molecular explanation why only a subset of small spines is capable to undergo synaptic plasticity.

Other studies also indicate the involvement of MMP-9 in modulation of dendritic spine structure. These findings demonstrate that effects of MMP-9 action substantially vary according to diverse experimental protocols. Non fully congruent effects of MMP-9 activity on spine structure can be explained by the different origin of MMP-9 in the system (exogenous application vs. endogenous release), dissimilarities in enzyme concentration used, a manner in which MMP-9 was applied (bath vs. local application), duration of MMP-9 activity influenced by endogenous inhibitors and maturity of neurons. For further discussion see: Dziembowska and Wlodarczyk 2012; Wiera et al., 2013 [64], [65].

### cLTP provokes MMP-dependent GluA1-AMPARs accumulation at dendritic spines

In the present study, we showed that cLTP increased the presence of GluA1-containing AMPARs at the dendritic spine surface. Our observations are consistent with previous studies that used fluorescence recovery after photobleaching (FRAP) or time-lapse imaging of AMPARs to demonstrate that AMPAR incorporation into synapses is accompanied by spine enlargement during cLTP [23], [59]. We also demonstrated that the blockade of MMP activity abolished GluA1-AMPAR accumulation at dendritic spines. This is an entirely novel observation. Furthermore, we showed that the cLTP-induced increase in GluA1 insertion into dendritic spines did not accompanied by an increase in spine number. The importance of our observations shall be considered in the context of the hypothesis that cLTP induces the insertion of GluA1-AMPARs into existing, but GluA-lacking and thus silent synapses and promotes spine maturation. It has been suggested that GluA1-containing receptors are inserted into dendritic spines during unsilencing of synapses upon LTP [66], [67]. Thus the relative changes in GLuA1 level are more pronounced on small, presumably previously silent synapses. However, we showed first time that extracellularly active MMPs facilitate GluA1-AMPAR accumulation at potentiated synapses and promote neuronal plasticity of local synapses during cLTP.

AMPARs have been shown to interact with a wide variety of intracellular and transmembrane proteins that control the targeting and signaling properties of AMPARs within the postsynaptic membrane [25], [68], [69]. However, the exact mechanisms by which MMPs adjust or control the surface expression of AMPARs at spines are poorly understood and likely to be complex [70], [71], [72], [73]. These mechanisms may involve different pathways and effectors. Indeed, the Ras and Rap guanosine triphosphatase (GTPase) family and its downstream effector cascades were shown to regulate AMPAR trafficking [70]. Also, synaptic activity stimulates a rapid MMP-dependent cleavage of intercellular adhesion molecule-5 (ICAM-5) [71]. The ectodomain ICAM-5 was found to stimulate an increase in phosphorylation and dendritic insertion of GluA1-AMPARs [72]. Furthermore, a growing body of evidence indicates that ephrins and Eph receptors, presumed substrates of MMPs, functionally interact with scaffolding and adapter proteins that regulate AMPA receptor trafficking and play a role in tethering AMPAR subunits in intracellular compartments (reviewed by [73]). Determining whether the regulation of Eph/ephrin interactions by MMPs is involved in AMPAR recruitment at spines upon cLTP will be interesting.

### cLTP immobilizes GluA1-AMPARs at dendritic spines: promotion by MMPs

Numerous studies have demonstrated the lateral movement of AMPARs into synapses during plasticity, but the mechanisms that control their synaptic incorporation remain poorly understood [74], [75], [76], [77], [78], [79], [80], [81] The issue of the involvement of ECM proteins in GluA1-AMPAR surface diffusion and immobilization at synapses during cLTP has not been resolved. An important finding from the current study was that extracellularly acting MMPs contributed to the regulation of GluA1-AMPAR trafficking into synapses during cLTP. We showed that cLTP stimulation of hippocampal neurons reduced the global and intrasynaptic mobility of GluA1-AMPARs, whereas the inhibition of MMP activity impaired GluA1-AMPAR immobilization. The redistribution of AMPARs from extrasynaptic to synaptic surface accounts for the fast exchange of desensitized receptors for naive functional receptors, thus increasing synaptic fidelity during fast repetitive stimulation [78]. Our findings indicate that GluA1-AMPAR trapping at synapses during cLTP is promoted by the proteolytic activity of MMPs; thus, MMPs modify synaptic strength during cLTP. Our results are consistent with a model in which synapse-specific slots capture passively diffusing GluA1-containing AMPARs from the nearby spine membrane during LTP [82]. We would like to suggest that the ability of MMPs to influence the distribution and the mobility of postsynaptic glutamatergic receptors might not mediated by a gross alterations of the extracellular matrix structure but rather by either releasing or revealing integrin binding ligands [83], [44], [55], [84]. The findings that gross ECM structure in not altered by MMP-9 was directly visualized using either hyaluronic binding protein or immunostaining against brevican [83]. Modifications of spine morphology and postsynaptic receptor content via integrin signalling appear to involve MMP-dependent cleavage of several proteins: β-dystroglycan, ICAM-5, neuroligin-1 [39,34,84,72, 8586,89].

Recently, Nair et al. [87] provided evidence that AMPARs are highly concentrated in nanodomains at dendritic spines. In the present study, we demonstrated that GluA1-containing AMPAR nanodomains are dynamic during cLTP and their appearance is regulated by MMP activity. In particular, the immobilization of AMPARs triggered the clustering of GluA1-AMPARs at synapses, which appeared to be MMP-dependent. Interestingly, a two-fold increase in the number of synaptic GluA1 clusters (>1 µm in diameter) after cLTP, was very similar in magnitude to the increase in the number spines expanding their heads. The inhibition of MMP activity by GM6001 prior to cLTP stimulation decreased the number of GluA1-AMPAR clusters at spines. Altogether, these data suggest that MMP activity facilitates neuronal transmission by regulating GluA1-AMPAR surface diffusion and clustering.

The ECM can restrict AMPAR movement, and the proteolytic degradation of ECM proteins and adhesion molecules was shown to generally facilitate AMPAR lateral diffusion [88]. In contrast, in our cLTP model, extracellular MMP activity was required for AMPAR surface expression, clustering, and immobilization at synapses during cLTP. This disparity may be attributable to the specific functions of MMP-9 at synapses. Several studies demonstrated that the effects of MMPs on spine morphology are mediated by the ability of MMP-9 to cleave specific ECM proteins and trigger integrin signaling signalling rather than to disrupt an overall structure of the ECM [42], [44]. Intercellular adhesion molecule-5 (ICAM-5), a negative regulator of spine maturation, was reported to be cleaved by MMP-9 in association with spine maturation after neuronal stimulation [71], [85] Additionally, [89] showed that MMP-9 was able to destabilize the presynaptic side and tune synaptic transmission through the proteolytic cleavage of neuroligin-1 at the postsynaptic side of glutamatergic synapses during plasticity.

In conclusion, our study reveals for the first time that pericellularly active proteinases (predominantly MMPs) are able to modify synaptic strength through AMPAR recruitment into synapses during plasticity. It shall also be noted that the morphological abnormalities of dendritic protrusions have been frequently associated with neuropsychiatric disorders, particularly those that involve cognitive deficits [7], [90], [91] and recent evidence implicates MMP-9 in aberrant synaptic plasticity and spine dysmorphology in neuropsychiatric disorders [92], [93], [94], [95]. Hence, our results open a new avenue of research on specific, mechanistic role of MMP-9 in the neuropsychiatric conditions, possibly contributing to novel treatments.

## Supporting Information

Figure S1 **cLTP mimics tetanus-induced LTP**. **A)** Representative single channel recordings of spontaneous electrical activity using a multielectrode array (MEA) are shown before and following cLTP induction. Chemical LTP enhances network activity and results in appearance of trains of bursts. **B–C)** Chemical LTP induces significant increases in both spiking and bursting activities.
(PDF)

Figure S2 **Spine density is not affected by cLTP.** The bar plot shows statistical analysis of spine density (number/µm) before, 10 and 40 min after cLTP stimulation. Under control conditions, the spine density was 0.82±0.08 (number of protrusions per µm^2^). With 40 min of cLTP, the spine density was 0.87±0.09. When the cLTP protocol was applied in the presence of 25 µM GM6001, the spine density was 0.83±0.07. Numbers represent mean ± SEM values.
(PDF)
