# Supplementary figures and images for: Synaptically Released Matrix Metalloproteinase Activity in Control of Structural Plasticity and the Cell Surface Distribution of GluA1-AMPA Receptors
Source: PLoS One. 2014 May 22;9(5):e98274. doi: 10.1371/journal.pone.0098274 (PMC4031140; doi:10.1371/journal.pone.0098274)

**A**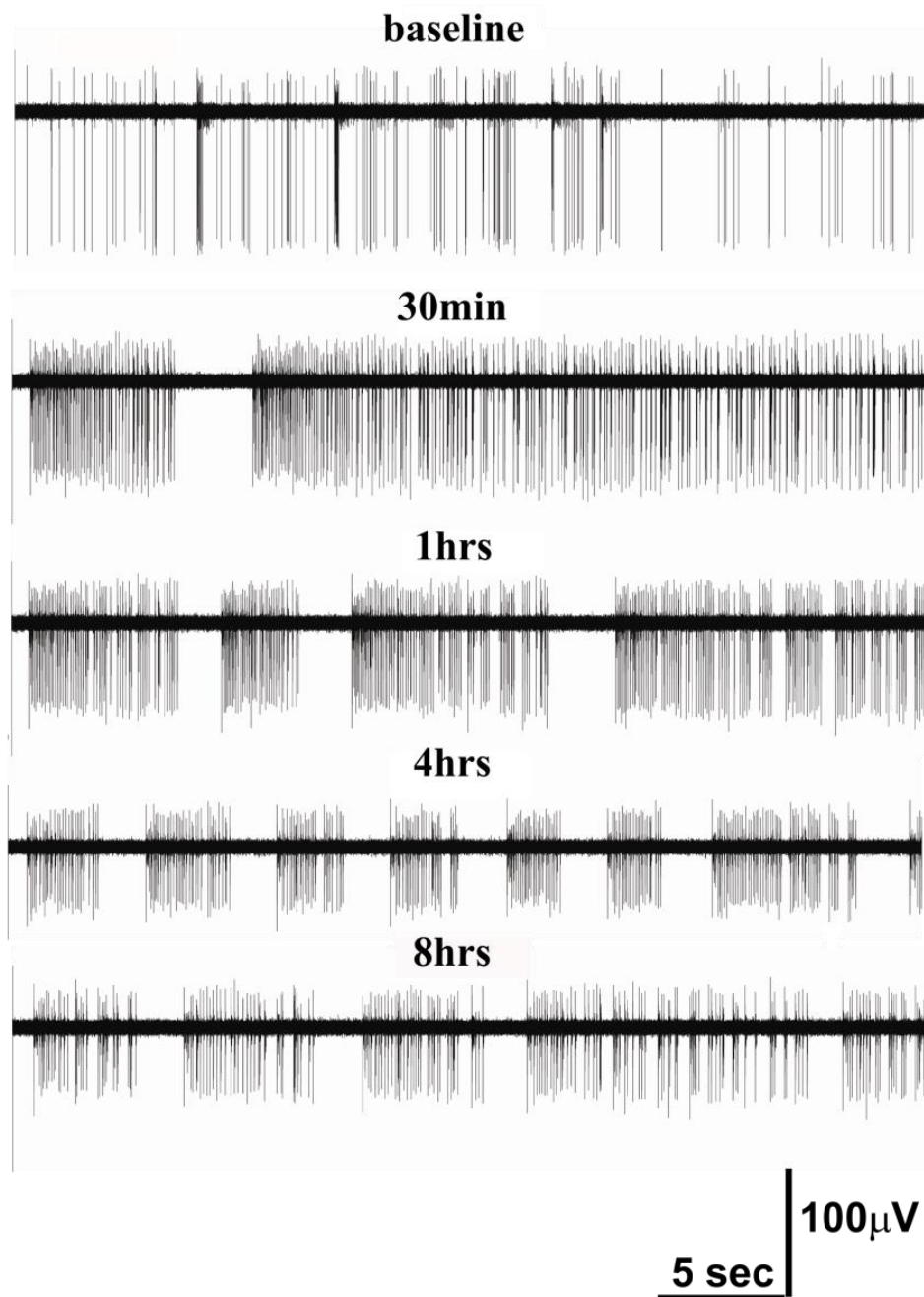**B**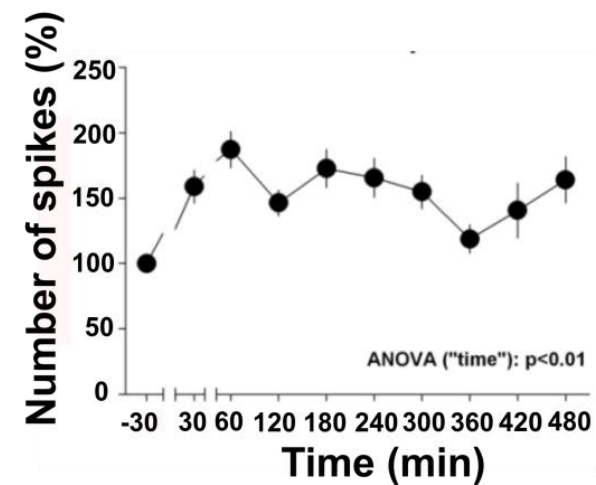**C**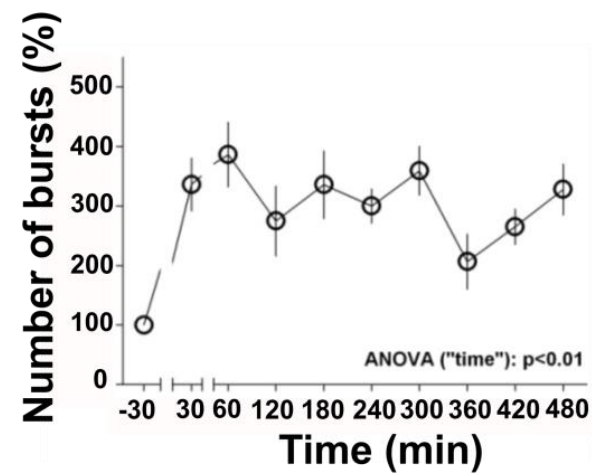**Fig. S1.**

Supplement: Figure S1 — cLTP mimics tetanus-induced LTP. A) Representative single channel recordings of spontaneous electrical activity using a multielectrode array (MEA) are shown before and following cLTP induction. Chemical LTP enhances network activity and results in appearance of trains of bursts. B–C) Chemical LTP induces significant increases in both spiking and bursting activities. (PDF) [file pone.0098274.s001.pdf]

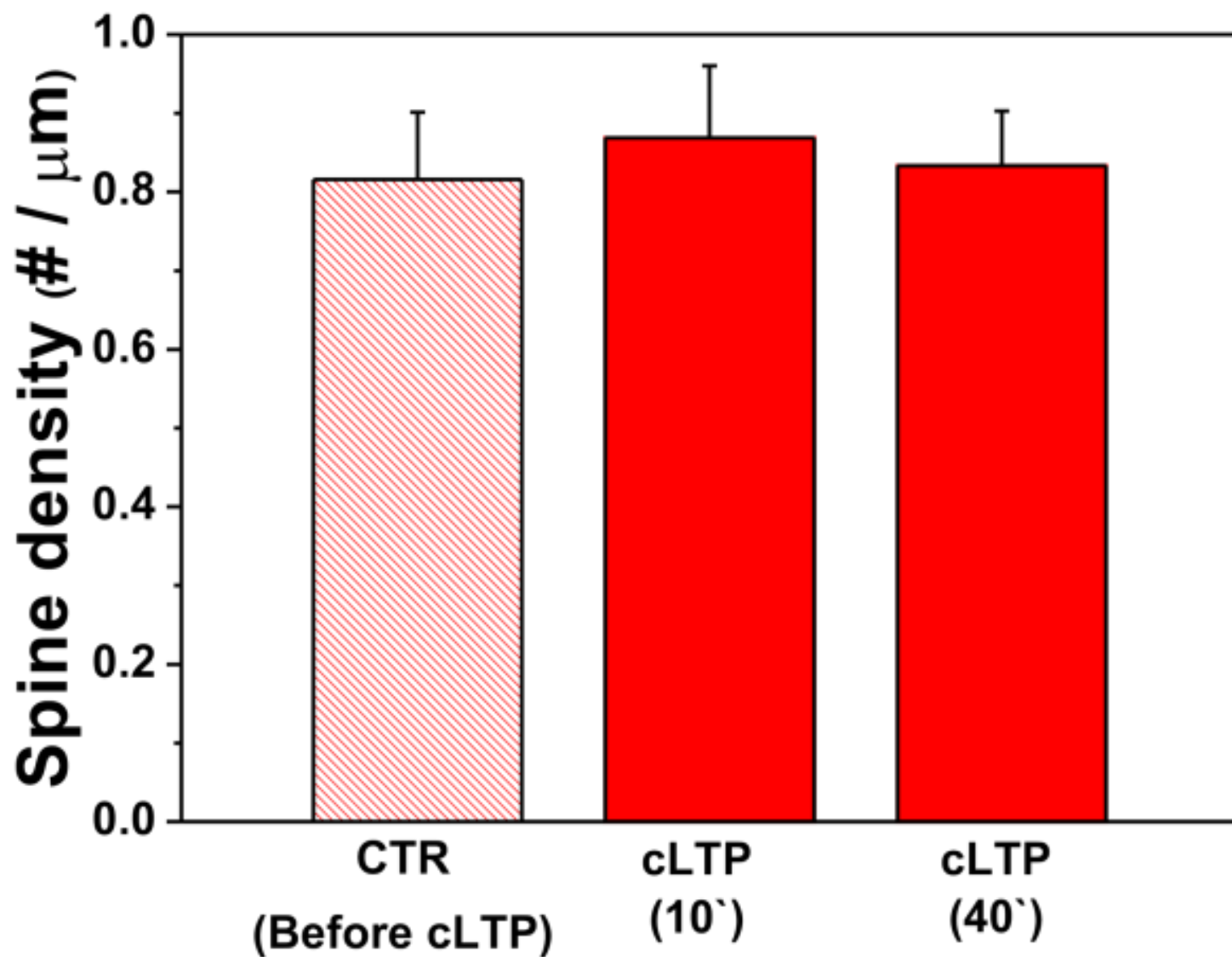

**Fig. S2.**

Supplement: Figure S2 — Spine density is not affected by cLTP. The bar plot shows statistical analysis of spine density (number/µm) before, 10 and 40 min after cLTP stimulation. Under control conditions, the spine density was 0.82±0.08 (number of protrusions per µm2). With 40 min of cLTP, the spine density was 0.87±0.09. When the cLTP protocol was applied in the presence of 25 µM GM6001, the spine density was 0.83±0.07. Numbers represent mean ± SEM values. (PDF) [file pone.0098274.s002.pdf]
